# Effects of a Proactive Driving Transition Class on Extending Safe Driving and Preparing for Life After Driving Cessation Among Older Drivers

**DOI:** 10.3390/geriatrics11020031

**Published:** 2026-03-16

**Authors:** Tsutomu Sasaki, Kyohei Yamada, Takeshi Yamakita, Naoto Sakuta, Hajime Yoshida, Takeshi Tominaga

**Affiliations:** 1 Department of Occupational Therapy, Hokkaido Chitose College of Rehabilitation, Chitose 0660055, Japan; k-yamada@chitose-reha.ac.jp; 2 Chitose-City Long-Term Care Prevention Center, Chitose 0660042, Japan; yamakita@chitose-houkatsu.jp; 3 Medical Corporation Shiseikai Chitose Hospital, Medical Center for Dementia, Chitose 0660067, Japan; d-center@siseikai.or.jp; 4 Chitose-City Kouyoudai Ward Community Comprehensive Support Center, Chitose 0660057, Japan; yoshida@chitose-renkei.com; 5 Chitose-City North Ward Community Comprehensive Support Center, Chitose 0660033, Japan; chitosekitashien@apricot.ocn.ne.jp

**Keywords:** older drivers, driving transition support, driving behavior, community mobility, proactive intervention

## Abstract

**Background/Objectives:** Driving cessation is associated with adverse health outcomes. Proactive support that extends safe driving while preparing for life after driving cessation has been emphasized, but empirical evidence remains limited. This study examined the effects of a proactive class for older drivers on awareness and behavior related to driving and mobility (Study 1) and on longitudinal changes in on-road driving behavior (Study 2). **Methods:** The proactive class was implemented as a municipal program, including information provision, training activities, group discussions, and optional on-road driving evaluations. Study 1 included 71 older drivers who attended the class at least five times annually and completed an anonymous questionnaire assessing perceived changes in awareness and behavior. Study 2 included 29 participants who completed standardized on-road driving evaluations at baseline and at a 1-year follow-up. Paired *t*-tests or Wilcoxon signed-rank tests with effect sizes were applied. **Results:** In Study 1, participants reported increased awareness of safe driving, greater confidence in continuing to drive, heightened risk perception, initiation of health-related behaviors, trial use of public transportation, and increased healthcare utilization, particularly ophthalmology visits. In Study 2, total scores on the on-road driving skill test improved significantly at follow-up (Cohen’s dz = 0.805). No significant changes were observed in individual on-road driving skill subitems, physical function, cognitive function, or daily functioning after correction for multiple comparisons, except for a reduction in driving simulator accidents. **Conclusions:** Participation in a proactive, continuous driving transition support class was associated with multidimensional behavioral changes and improved on-road driving performance among older drivers, potentially contributing to safer mobility and healthier aging.

## 1. Introduction

Driving is an essential means of mobility for maintaining daily life and health among older adults. Beyond enabling transportation to destinations, driving also plays an important role in supporting autonomy and self-esteem in later life [1]. Reasons for driving cessation among older adults can be broadly classified into health-related and non-health-related factors [2,3]. Regardless of the underlying reason, driving cessation has been consistently associated with an increased risk of health deterioration [4,5,6].

In recent years, increasing attention has been paid to the importance of driving transition support aimed at minimizing the adverse health effects associated with driving cessation. Such support focuses on preparing older adults for life after driving cessation and enabling them to maintain healthy and active lives. A recent systematic review on driving transition support highlighted several key components, including preparing for driving cessation in advance, developing concrete mobility plans, involving multiple professionals (including healthcare providers) in the decision-making process, and establishing systems that respect the individual’s preferences and autonomy [7].

To date, only three studies have empirically examined the effectiveness of driving transition support interventions. Two studies evaluated educational programs for individuals with dementia and their families [8,9], while one study investigated the effects of a driving cessation transition intervention targeting older adults who had recently stopped driving or planned to cease driving within one year [10]. That study demonstrated improvements in outing frequency, use of non-driving transportation modes, and self-efficacy regarding post-driving cessation mobility immediately after a six-week intervention; however, these effects were no longer evident at the three-month follow-up [10]. These findings suggest that short-term, intensive interventions alone may be insufficient and underscore the need for sustained, ongoing support.

Another important issue highlighted by Liddle et al. (2014) [10] is the difficulty of identifying, at the community level, older adults who have just ceased driving or who plan to stop driving within a year. Consequently, limiting support programs exclusively to individuals with imminent or confirmed driving cessation may not be feasible. Instead, it may be more appropriate to target all older drivers, providing education during the period when they are still driving to both prolong safe driving duration and facilitate preparation for life after driving cessation. This educational approach is consistent with evidence indicating that acceptance of driving cessation often requires time [11] and with prior research emphasizing the importance of reducing psychological conflict during the transition process [12]. From this perspective, such an approach can be considered a valid and appropriate form of driving transition support.

Against this background, we have been implementing a proactive class as part of a municipal initiative in the city of Chitose since July 2021. This class provides programs aimed at extending the period of safe driving as well as programs designed to support preparation for life after driving cessation. The present study examined the effects of this class from two perspectives. First, we conducted a cross-sectional analysis to investigate the impact of the class on awareness and behaviors related to mobility, including automobile use and public transportation. Second, we conducted a longitudinal analysis to examine the effects of the class on driving behavior.

## 2. Materials and Methods

### 2.1. Description of the Class

The class was conducted in the city of Chitose, Hokkaido, Japan. The city of Chitose has a population of approximately 100,000 residents, with an aging rate of about 24%. The city experiences heavy snowfall from January to March. The primary modes of daily transportation within the city, other than private automobiles, are buses and taxis; however, service reductions and driver shortages have become significant local challenges.

The class is a proactive program designed to extend the period of safe driving among older drivers while simultaneously supporting preparation for life after driving cessation. The program is implemented through collaboration among multiple professionals (occupational therapists, public health nurses, mental health social workers, and social workers) and multiple organizations, including community comprehensive support centers, preventive care centers, medical institutions, municipal administrative offices, and driving schools.

The class is offered as a single annual package from April to December, with sessions held once per month. Each session lasts 90 min, for a total of nine sessions per year (excluding the snow season from January to March). Eligible participants are older drivers aged 65 years or older who reside in the city of Chitose. Participants are recruited through the municipal public relations magazine, social networking services, and posted notices at commercial facilities and administrative offices within the city. In principle, interested individuals register for participation in March of each year and are encouraged to attend sessions monthly; however, individuals wishing to join after April are also accepted. Participation is free of charge.

The class covers content related to extending the safe driving period as well as preparation for life after driving cessation. Each session consists of three components. The first component focuses on providing information related to driving and mobility behaviors, with the aim of ensuring that participants have accurate knowledge regarding community mobility. This component lasts approximately 10 min and includes topics such as the driver’s license renewal system, traffic laws, safe driving practices and accident prevention strategies, local transportation services and mobility subsidies, and the relationships between driving and conditions such as dementia, ophthalmologic diseases, and lifestyle-related diseases. The second component aims to enhance participants’ awareness of abilities related to driving and lasts approximately 30 min. This component includes stretching exercises, cognitive training, vision training, and hazard perception training. Participants are also provided with guidance on how to continue these exercises at home. The third component consists of group-based activities designed to help participants reflect on their current driving status and develop an image of life after driving cessation. This component lasts approximately 50 min. Topics discussed include strategies for safe driving, identification of locations within participants’ daily travel routes where driving feels hazardous, precautions when driving during the snowy season, anticipated changes in daily life after driving cessation, use of alternative transportation options within the community, and perceived inconveniences and benefits associated with driving cessation. After group discussions, the content is shared among all participants. For each class session, one theme is addressed within each component.

In addition to the class sessions, participants are offered a free on-road driving evaluation conducted by certified driving instructors from a driving school on a separate day. This evaluation is intended to promote self-reflection on individual driving ability and lasts approximately 50 min.

From July 2021 through December 2025, a total of 41 class sessions were held. During this period, 86 individuals registered for the program, with a cumulative total of 718 participant attendances.

### 2.2. Study Design and Participants

#### 2.2.1. Overall Study Design

This study consisted of two components: Study 1, a cross-sectional survey examining changes in awareness and behavior related to driving and mobility after class participation, and Study 2, a longitudinal pre–post evaluation assessing changes in on-road driving performance over a one-year period.

#### 2.2.2. Overall Participant Flow

A CONSORT-style flowchart illustrating participant inclusion and attrition across Study 1 and Study 2 is provided in the Supplementary Materials.

### 2.3. Study 1. Effects on Awareness and Behavior

#### 2.3.1. Participants

Participants were older drivers who attended the class at least five times in a single fiscal year between 2022 and 2025. A total of 71 participants met this criterion and were included in the analysis.

#### 2.3.2. Procedures

At the end of the class session during which participants reached their fifth attendance within a given year, they were asked to complete an anonymous questionnaire. The questionnaire assessed perceived changes in awareness and behavior related to driving and mobility. The full questionnaire used in Study 1, originally developed in Japanese and consisting of 11 items derived from the 2021 pilot study, is provided in Supplementary Material S1. Multiple responses were permitted. The questionnaire items consisted of 11 items identified through interviews with participants in the 2021 program year:

(1) Became more conscious of safe driving;
(2) Felt more confident in continuing to drive;
(3) Became aware of eye movements while driving;
(4) Became more attentive to news about older drivers;
(5) Became aware of the need for cognitive training for driving;
(6) Started doing stretching or exercise;
(7) Developed a greater interest in traffic laws and the driver’s license system;
(8) Started thinking about when to return the driver’s license;
(9) Began improving lifestyle habits (e.g., diet and daily routines);
(10) Tried using public transportation to visit destinations usually reached by car;
(11) Started visiting a new medical department.

An additional open-ended option (“Other [free-text response]”) was provided; however, no participants selected this option, and it was therefore excluded from the analysis.

#### 2.3.3. Measures and Analysis

For each questionnaire item, the number of participants who selected the item was counted. The total number of respondents for each item was used as the outcome measure.

### 2.4. Study 2. Effects on Driving Behavior

#### 2.4.1. Participants

Among 75 individuals who underwent an on-road driving evaluation conducted by certified driving instructors between fiscal years 2021 and 2025, 29 participants who completed driving evaluations at both baseline and the 1-year follow-up were included in the analysis. The mean age of the participants was 74.1 ± 3.8 years (range: 67–82 years), including 15 men and 14 women. Baseline characteristics of the participants are presented in Table 1.

#### 2.4.2. Procedures

Participants underwent a 50 min on-road driving evaluation on a day separate from the class sessions. The on-road driving evaluations were conducted by certified driving instructors affiliated with licensed driving schools in Japan. These instructors were not occupational therapists. The purpose of the on-road evaluation was not to determine overall fitness to drive but to observe on-road driving behavior and provide feedback focusing on tactical and operational aspects of driving performance. Strategic aspects of driving, such as decisions regarding driving continuation or cessation, were not assessed during the on-road evaluation. The evaluation consisted of a 25 min drive within the city, followed by individualized feedback from a certified driving instructor regarding driving performance. Based on this feedback, participants then completed an additional 15 min drive within the city. After the on-road evaluation, individualized feedback was provided by certified driving instructors following a standardized structure based on predefined evaluation domains (e.g., safety checks, speed control, stop sign compliance, and turning behavior). The feedback included identification of observed issues, explanation of appropriate driving behaviors, and practical safety-oriented advice. While the structure and evaluation domains of the feedback were consistent across participants, the specific content was tailored to each individual’s driving performance. The driving route and the locations at which feedback was provided were standardized across all participants. Evaluations were conducted between 14:00 and 14:50, and the total driving distance was approximately 10 km. The follow-up driving evaluation, conducted one year later, followed the same protocol. Four certified driving instructors conducted the evaluations. Fifteen participants were evaluated by the same instructor at both baseline and follow-up. After completing the driving evaluation, participants underwent assessments of physical and cognitive function, daily functioning, and off-road driving ability on a separate day at the authors’ affiliated institution.

#### 2.4.3. Measures and Analysis

Driving behavior was assessed using the total score and the number of identified errors for each evaluation item on the standardized on-road driving skill test score sheet designated by the Public Safety Commission. This standardized on-road driving skill test is based on an evaluation framework routinely used at licensed driving schools in Japan under the supervision of the Public Safety Commission. The evaluation items and deduction-based scoring procedures are standardized according to publicly available official guidelines, and the same framework is consistently applied nationwide. The total score was calculated using a deduction system starting from 100 points. Eleven major deduction categories were defined, each containing multiple subitems: driving posture, safety checks, braking, steering control, vehicle positioning, lane discipline, lane changes, straight driving and turning, pedestrian protection, maximum speed, and test termination. Within these categories, individual deduction items were weighted differently (e.g., 20-, 10-, or 5-point deductions), reflecting the severity of each driving error. No participants in the present study received deductions for lane discipline; therefore, this category was excluded from the analysis. Among the test termination subitems, all applicable cases involved failure to stop at a stop sign; other subitems were therefore excluded from the analysis.

Three measures were used for physical and cognitive function. Physical function was assessed using the Short Physical Performance Battery (SPPB) [13], which consists of balance, gait speed, and chair stand components and is scored on a 12-point scale, with higher scores indicating better physical function. Cognitive function was assessed using the Trail Making Test—Japanese version (TMT-J) [14], which includes Part A (connecting numbers sequentially) and Part B (alternating between numbers and letters); completion times for each part were recorded. Global cognitive function was assessed using the Mini-Mental State Examination (MMSE) [15], which evaluates domains such as orientation, memory, calculation, praxis, and copying, with scores ranging from 0 to 30 (higher scores indicate better cognitive function). Daily functioning was assessed using the Kihon Checklist [16]. Participants responded “yes” or “no” to 25 items across seven domains: activities of daily living, physical function, nutrition, oral function, social withdrawal, cognitive function, and depressive mood. Scores range from 0 to 25, with lower scores indicating better daily functioning. Off-road driving ability was assessed using a driving simulator (Safety Navi; Honda Motor Co., Ltd., Tokyo, Japan). Outcome measures included reaction times on driving response tasks (simple reaction time, selection reaction time, and complex reaction time), the number of collisions during driving tasks, and the proportion of overspeeding (defined as the ratio of distance driven above the speed limit to the total driving distance). All measures were compared between baseline and the 1-year follow-up.

In the statistical analysis, the on-road evaluation total score was defined a priori as the primary outcome. All other variables were treated as secondary outcomes and grouped into conceptual families: daily functioning (Kihon Checklist, 1 item), physical function (SPPB, 1 item), cognitive function (MMSE, TMT-A, TMT-B; 3 items), driving simulator cognitive measures (simple reaction time, selection reaction time, complex reaction time; 3 items), driving simulator operational measures (accidents, overspeeding; 2 items), and on-road driving skill subitems (10 items). When assumptions of normality and homogeneity of variance were met, paired *t*-tests were applied to compare baseline and follow-up values, and effect sizes (Cohen’s dz) with 95% confidence intervals were calculated. Cohen’s dz values were interpreted as follows: <0.20, negligible; 0.20–0.49, small; 0.50–0.79, medium; and ≥0.80, large [17]. When these assumptions were not met, the Wilcoxon signed-rank test was used, with 95% confidence intervals estimated using the Hodges–Lehmann estimator and effect sizes calculated using Cliff’s delta. Cliff’s delta values were interpreted as follows: <0.147, negligible; 0.147–0.329, small; 0.330–0.473, medium; and ≥0.474, large [18]. A post hoc power analysis for the primary outcome (on-road evaluation total score) indicated that the sample size of 29 participants was sufficient to detect medium-to-large effect sizes in paired comparisons. To account for multiple comparisons among secondary outcomes, the Holm correction was applied within each family. The significance level was set at 5%. All statistical analyses were performed using R version 4.4.3.

## 3. Results

### 3.1. Study 1. Effects on Awareness and Behavior

**Figure 1:**
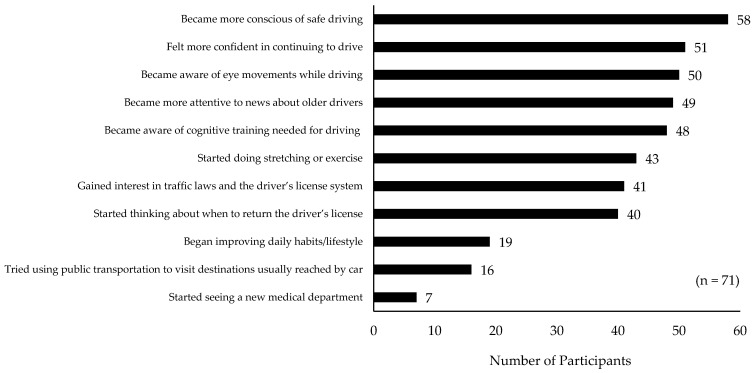
Effect of the class on awareness and behavior (multiple responses allowed).

Among the 71 participants included in the analysis, the most frequently reported change was becoming more conscious of safe driving (*n* = 58). This was followed by becoming aware of eye movements while driving (*n* = 51), becoming more attentive to news about older drivers (*n* = 50), and developing a greater interest in traffic laws and the driver’s license system (*n* = 49). Feeling more confident in continuing to drive was reported by 48 participants, and increased awareness of the need for cognitive training for driving was reported by 43 participants. With respect to health-related behaviors, 41 participants reported starting stretching or exercise, and 19 reported improving lifestyle habits such as diet and daily routines. Forty participants reported beginning to think about when to return their driver’s license. In terms of mobility behavior, 16 participants reported trying to use public transportation to visit destinations they usually reached by car. Seven participants reported visiting a new medical department; all of these visits were to ophthalmology clinics (Figure 1).

### 3.2. Study 2. Effects on Driving Behavior

**Figure 2:**
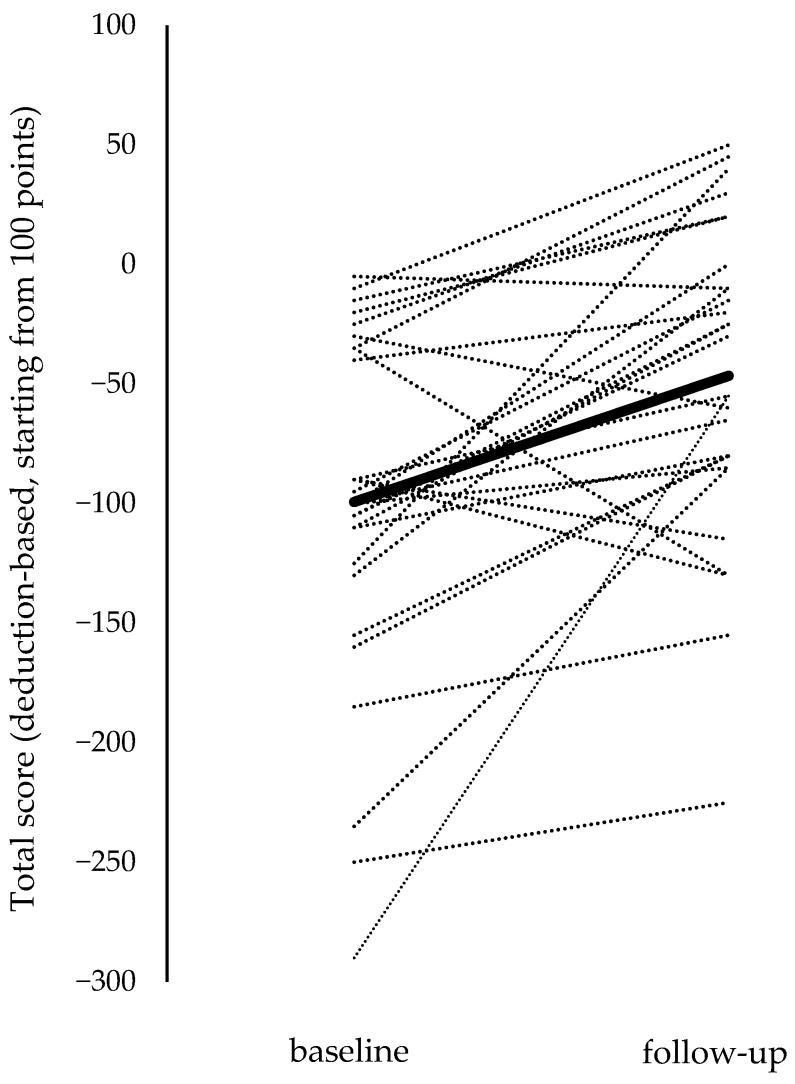
Changes in total scores on the on-road driving skill test. Dotted lines represent individual participants, and the bold line represents the mean change across all participants.

The total score on the on-road driving skill test improved significantly at follow-up compared with baseline (*p* < 0.001, Cohen’s dz = 0.805) (Figure 2).

Regarding individual evaluation items, no significant changes were observed in the number of identified errors for any on-road driving skill subitems after Holm correction. Similarly, no significant differences were observed in physical function, cognitive function, or daily functioning between baseline and follow-up. Among driving simulator measures, the number of accidents during the simulator task decreased significantly at follow-up, and this difference remained statistically significant after Holm correction (Holm-adjusted *p* = 0.01, Cliff’s delta = −0.313) (Table 2).

## 4. Discussion

This study examined the effects of a proactive class for older drivers on awareness and behavior (Study 1) as well as on on-road driving behavior (Study 2). The findings indicate that participation in the class was associated with changes in self-awareness related to driving, risk perception, health-related behaviors, and trial use of alternative transportation modes, and that, longitudinally, participation was also associated with improvements in on-road driving skill test performance. These results suggest that the dual focus of the class—extending the period of safe driving while preparing for life after driving cessation—may facilitate multidimensional behavioral change.

### 4.1. Impact on Awareness and Self-Perceived Behavioral Change (Study 1)

In Study 1, many participants reported increased interest in driving-related information and enhanced self-efficacy regarding continued driving. Information provision during the class, activities aimed at increasing awareness of driving-related abilities, and feedback obtained through on-road evaluations likely contributed to improved self-understanding. Furthermore, increased sensitivity to social and institutional information—such as paying more attention to news about older drivers, developing interest in traffic laws and the driver’s license system, and beginning to consider the timing of driver’s license return—aligns with prior research emphasizing the importance of respecting individual preferences and autonomy in the driving transition process [7].

Importantly, the effects of the class extended beyond driving behavior to health-related and healthcare-seeking behaviors. A proportion of participants reported initiating stretching or exercise and improving lifestyle habits, suggesting that the exercise and cognitive training component of the class may have contributed to behavioral change in daily life. In addition, the observed increase in ophthalmology visits indicates that information provided about the relationship between visual function and driving may have prompted appropriate healthcare utilization. This is particularly noteworthy given that early detection of glaucoma—often asymptomatic in its early stages—is essential for safe driving [19]. Moreover, the reported trial use of public transportation is consistent with the class objective of encouraging experimentation with alternative mobility options during the driving continuation phase rather than immediately before driving cessation. Given the vulnerability of local transportation systems in the city of Chitose, early exposure to non-driving transportation modes may help mitigate perceived inconvenience after driving cessation. Through group-based activities and dialogue with peers, the class also appears to provide opportunities for participants to update their perspectives on driving and community mobility, consistent with prior findings on peer interaction and reflective learning [20].

### 4.2. Longitudinal Changes in On-Road Driving Skills (Study 2)

In Study 2, total scores on the on-road driving skill test improved significantly at the 1-year follow-up, whereas no significant changes were observed in the number of identified errors for individual driving skill subitems after adjustment for multiple comparisons. This apparent discrepancy can be explained by the weighted scoring system used in the on-road evaluation. Although the total number of errors did not change, reductions in errors associated with higher-weight deduction items may have contributed disproportionately to improvements in the total score. Accordingly, the observed improvement likely reflects qualitative changes in driving performance, such as the mitigation of more critical driving errors, rather than a simple reduction in the frequency of minor errors. Previous research has shown that driving ability in older adults tends to decline over time due to changes in health status [21]. Despite the relatively short follow-up period in the present study, improvements in on-road driving performance were observed. This finding suggests that continued learning through the proactive class, together with individualized feedback from certified driving instructors, may have positively influenced actual driving behavior. In addition, region-specific program content, such as group discussions on winter driving and hazard perception training, may have been translated into safer on-road driving practices.

In contrast, no change was observed in errors related to failure to stop at stop signs. In Japan, failure to comply with stop signs is the most common traffic violation [22], and compliance with stop signs may be difficult to improve through conscious effort alone. One possible explanation for this finding is the strict evaluation criteria applied by driving instructors: even when drivers slowed down and visually confirmed safety, failure to come to a complete stop was recorded as an error. Such criteria may have limited the sensitivity of the assessment to detect behavioral changes commonly observed in everyday driving. This finding suggests that stop sign performance may be less responsive to short-term educational interventions and should be interpreted cautiously within the context of assessment characteristics. In addition, stop sign-related errors were relatively infrequent overall, and no consistent pattern of repeated failures by the same individuals was observed. This finding further supports the interpretation that stop sign behavior may represent an overlearned task for most participants. However, the present evaluation did not distinguish between different types of stop sign errors, such as complete failure to notice a sign versus incomplete stopping, nor was it designed to examine individual-level error repetition. These aspects warrant further investigation in future studies focusing on fitness-to-drive assessment. Furthermore, stop sign performance may also be influenced by environmental characteristics, including the placement, size, and visibility of the signs, which affect visual and divided attention demands. In the present study, stop sign locations were standardized and did not intentionally include visually challenging configurations. Therefore, the results should be interpreted within the context of relatively uniform environmental conditions.

Although no significant changes were observed in physical function (SPPB), cognitive function (TMT-J, MMSE), daily functioning (Kihon Checklist), or off-road driving ability, a significant reduction was observed in the number of accidents during the driving simulator task. This finding suggests that the class may not have improved underlying functional capacity per se, but rather altered hazard perception and attentional behavior within existing functional limits. The class can thus be characterized as an intervention that supports safe driving through accurate self-understanding and adaptive behavior, rather than through direct remediation of functional decline. From this perspective, the class may influence metacognitive abilities related to driving [23]. In addition, age may play an important role in moderating the effects of proactive driving transition support. Although the present longitudinal analysis did not examine age-stratified effects due to the limited sample size, future studies with larger cohorts should investigate whether program effects differ between younger-old and older-old drivers.

The fact that the class is provided as a year-long package is also noteworthy. Previous studies have reported that the effects of short-term, intensive interventions diminish within three months [10]. In contrast, the present class combines nine sessions of continuous learning with optional on-road evaluations, which may have contributed to the maintenance and reinforcement of awareness and behavioral changes. Continued participation may also provide opportunities to initiate gradual driving regulation or self-regulation behaviors [24], supporting a shift from interventions focused solely on the period immediately before or after driving cessation toward earlier, more comprehensive engagement.

### 4.3. Limitations

Several limitations should be acknowledged. First, Study 1 employed a cross-sectional design without a control group and relied on self-reported changes in awareness and behavior; therefore, causal relationships between class participation and observed changes cannot be established. In addition, the questionnaire used in Study 1 was developed for exploratory purposes based on interviews conducted in 2021 and has not undergone formal psychometric validation. Therefore, the results should be interpreted as participants’ self-perceived changes rather than as standardized measures of awareness or behavior. Selection bias is also possible, as older adults with greater interest in safe driving or health behaviors may have been more likely to continue participating. Additionally, because participants voluntarily enrolled in the class after reviewing its content, the influence of attention bias cannot be excluded. Second, Study 2 included only 29 participants who completed both baseline and follow-up on-road evaluations, and these individuals may differ from those who underwent only a single evaluation or discontinued follow-up. Moreover, although the on-road evaluation employed a weighted deduction system, the present analysis focused on the number of identified errors rather than the distribution of error severity. Due to the limited sample size and low frequency of high-weight deduction events, the study was underpowered to statistically examine changes in specific weighted error categories. Future studies with larger samples and accumulated events are needed to more precisely evaluate changes in error severity patterns. Accordingly, the study may have been underpowered to detect small effects in secondary outcomes. Although the driving route was standardized, repeated exposure to the same approximately 10 km course may have introduced familiarity or learning effects. Furthermore, although the on-road driving route and evaluation timing were standardized, on-road assessments are inherently influenced by real-world traffic conditions. While no evaluations were discontinued due to major external disruptions, minor variations in surrounding traffic could not be fully controlled, which may have affected driving performance. Third, because four driving instructors conducted the evaluations and approximately half of the participants were assessed by different instructors at baseline and follow-up, inter-rater variability cannot be fully ruled out. Fourth, this study did not track actual driving cessation timing or long-term health outcomes (e.g., depressive symptoms, decline in daily functioning, or healthcare and long-term care utilization); therefore, the medium- to long-term effects of class participation on safe driving duration and health outcomes remain unclear.

### 4.4. Future Directions

Future research should employ prospective study designs with control groups to examine associations between class participation (including participation frequency), duration of driving continuation, post-cessation inconvenience, and health outcomes. In addition, randomized controlled trials, where feasible, will be important to more rigorously evaluate the causal effects of proactive driving transition programs. Based on the present findings, the development of modules targeting specific driving behaviors—such as stop-sign compliance and speed management—and the introduction of tailored feedback based on individual driving evaluation results should also be considered. Furthermore, the applicability and external validity of the class framework should be examined not only in municipalities with characteristics similar to the city of Chitose (e.g., heavy snowfall, reduced public transportation services) but also in urban areas with more accessible public transportation systems.

## 5. Conclusions

This study suggests that a proactive driving transition support class for older drivers may be associated with changes in driving-related awareness, health-related and healthcare-seeking behaviors, trial use of alternative transportation modes, and improvements in on-road driving skills. These findings extend beyond conventional support targeting individuals with imminent or confirmed driving cessation and underscore the importance of establishing continuous support spanning the driving continuation phase through post-cessation life. Such an approach may contribute to community-based efforts to balance safe mobility and health maintenance among older adults.

## Figures and Tables

**Table 1:** Characteristics of the participants.

	Baseline	Follow-Up
number (male/female)	29 (15/14)	29 (15/14)
Age ± standard deviation. (min–max, median)	74.1 ± 3.8 (67–82, 74)	75.3 ± 3.7 (68–83, 75)
living with family (yes/no)	20/9	20/9
driving license history		
21–30 years	2	
31–40 years	1	
>41 years	26	
driving frequency		
6–7 days per week	19	13
3–5 days per week	9	14
1–2 days per week	1	2
driving duration		
>5 h per week	6	7
3–5 h per week	12	10
1–3 h per week	9	9
30 min–1 h per week	1	2
<30 min per week	1	1
traffic accidents in the past year * (yes/no)	4/25	5/24
traffic violations in the past year (yes/no)	1/28	2/27

* Includes accidents not involving the police (e.g., minor collisions in private parking areas), no-fault accidents, and unavoidable accidents.

**Table 2:** Comparison of baseline and follow-up measures of daily function, physical function, cognitive function, and off-road driving abilities.

	Baseline Mean ± Sd (Min–Max, Median)	Follow-Up Mean ± Sd (Min–Max, Median)	*p* Value	Holm-Adjusted *p* Values	95% Confidence Interval (Difference)			Effect Size
Kihon Checklist (/25) (points)	3.0 ± 1.7 (0–8, 3)	2.7 ± 1.9 (0–7, 3)	0.422		−1.0	-	0.5	−0.098 (δ)
SPPB (/12) (points)	11.2 ± 1.0 (9–12, 12)	11.2 ± 1.0 (9–12, 12)	1.000	1.000	−0.5	-	1.0	0.020 (δ)
MMSE (/30) (points)	28.6 ± 1.5 (25–30, 29)	28.5 ± 1.3 (25–30, 29)	0.505	1.000	−1.0	-	1.0	−0.145 (δ)
TMT-J (s)								
part-A (s)	47.4 ± 15.3 (23.5–72.4, 45.3)	48.5 ± 17.2 (22.6–87.5, 46.7)	0.684	1.000	−4.4	-	6.6	0.076 (dz)
part-B (s)	102.5 ± 45.5 (49.7–227.1, 93.8)	112.0 ± 73.6 (44.9–405.4, 94.3)	0.665	1.000	−11.6	-	9.1	0.024 (δ)
DS response task (s)								
Simple RT (s)	0.4 ± 0.04 (0.3–0.5, 0.37)	0.4 ± 0.04 (0.3–0.5, 0.37)	0.629	1.000	−0.007	-	0.011	0.091 (dz)
Selection RT (s)	0.8 ± 0.1 (0.6–1.0, 0.76)	0.8 ± 0.1 (0.6–1.0, 0.73)	0.174	0.694	−0.045	-	0.008	−0.259 (dz)
Complex RT (s)	1.0 ± 0.1 (0.8–1.4, 0.98)	1.0 ± 0.1 (0.8–1.4, 0.97)	0.369	1.000	−0.1	-	0.0	−0.170 (dz)
DS driving task								
accident (number) *	2.1 ± 1.8 (0–7, 2)	1.2 ± 1.1 (0–7, 1)	0.002	0.010	−2.0	-	−0.5	−0.313 (δ)
overspeeding (%)	8.6 ± 8.1 (0–29.5, 7.4)	9.2 ± 7.5 (0–28.2, 9.0)	0.741	1.000	−2.7	-	3.7	0.062 (dz)
On-road driving skill test								
total score (points) *	−99.3 ± 73.1 (−290–5, −100)	−46.6 ± 64.8 (−225–50, −30)	0.0002		27.8	-	77.7	0.805 (dz)
driving posture (count)	0.1 ± 0.3 (0–1, 0)	0.1 ± 0.3 (0–1, 0)	1.000	1.000	−1	-	1	0.000 (δ)
safety check (count)	10.3 ± 3.1 (5–18, 9)	8.6 ± 3.7 (2–16, 9)	0.045	0.315	−3.4	-	−0.04	−0.390 (dz)
braking (count)	0.2 ± 0.5 (0–2, 0)	0.03 ± 0.2 (0–1, 0)	0.037	0.295				−0.139 (δ)
steering (count)	0.7 ± 1.1 (0–4, 0)	1.0 ± 1.4 (0–1, 1)	0.480	1.000	−1.0	-	2.00	0.106 (δ)
vehicle control (count)	3.3 ± 2.1 (0–8, 3)	2.0 ± 1.5 (0–6, 2)	0.019	0.170	−2.3	-	−0.2	−0.463 (dz)
lane change (count)	2.3 ± 2.4 (0–9, 2)	1.7 ± 1.8 (0–6, 1)	0.200	1.000	−1.4	-	0.3	−0.244 (dz)
straight driving and turning (count)	1.2 ± 1.2 (0–4, 1)	0.8 ± 1.0 (0–4, 1)	0.177	1.000	−0.9	-	0.2	−0.257 (dz)
pedestrian protection (count)	0.03 ± 0.2 (0–1, 0)	0	1.000	1.000				−0.034 (δ)
overspeeding (count)	1.4 ± 1.2 (0–4, 1)	0.7 ± 1.1 (0–4, 0)	0.011	0.115	−2.0	-	−0.5	−0.353 (δ)
failure to stop at a stop sign (count)	0.7 ± 0.8 (0–3, 1)	0.7 ± 0.7 (0–2, 1)	0.704	1.000	−1.0	-	0.5	−0.023 (δ)

Values are presented as mean (SD) or median (interquartile range), as appropriate. Statistically significant differences are indicated by bold text and an asterisk (*). SPPB: Short Physical Performance Battery; MMSE: Mini-Mental State Examination; TMT-J: Trail Making Test—Japanese version; DS: driving simulator; RT: reaction time. Effect sizes are reported as Cohen’s dz (dz) for parametric tests and Cliff’s delta (δ) for non-parametric tests. The criteria for Cohen’s dz were as follows: values < 0.20 were considered negligible; 0.20–0.49, small; 0.50–0.79, medium; and ≥0.80, large. The criteria for Cliff’s delta were values < 0.147, negligible; 0.147–0.329, small; 0.330–0.473, medium; and ≥0.474, large.

## Data Availability

The data used to support the findings of this study will be available from the authors upon reasonable request.

## Supplementary Materials

The following supporting information can be downloaded at: https://www.mdpi.com/article/10.3390/geriatrics11020031/s1, Supplementary Material S1: CONSORT-style flowchart illustrating participant inclusion and attrition across Study 1 and Study 2, including reasons for exclusion at each stage; Material S2: The Questionnaire used in Study 1.
