# Supplementary figures and images for: Effects of a Proactive Driving Transition Class on Extending Safe Driving and Preparing for Life After Driving Cessation Among Older Drivers
Source: Geriatrics (Basel). 2026 Mar 16;11(2):31. doi: 10.3390/geriatrics11020031 (PMC13010717; doi:10.3390/geriatrics11020031)

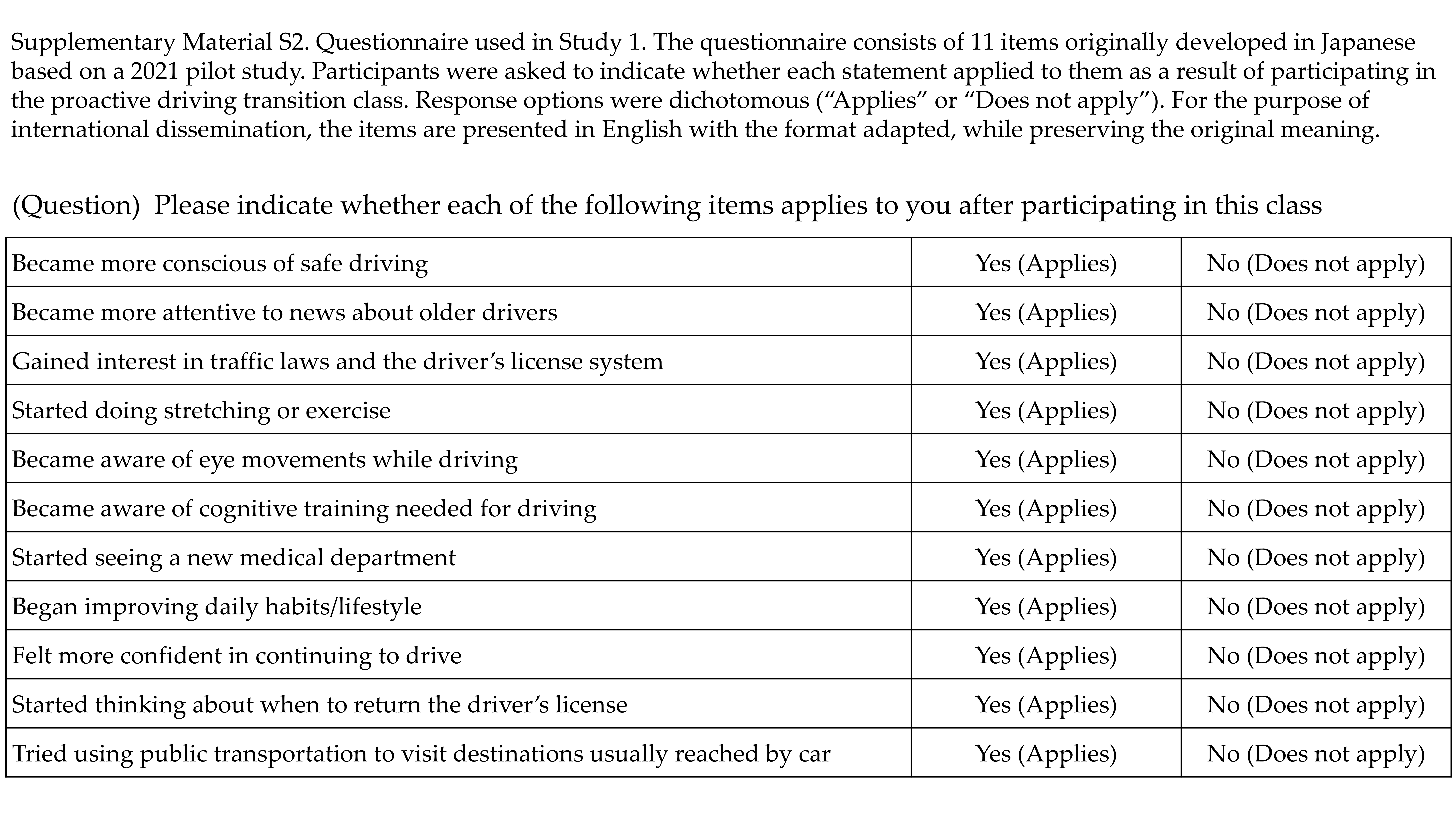

Supplement: Supplementary file 1 [file geriatrics-11-00031-s001.zip › S1&2_revised3/S2_revised3.png]

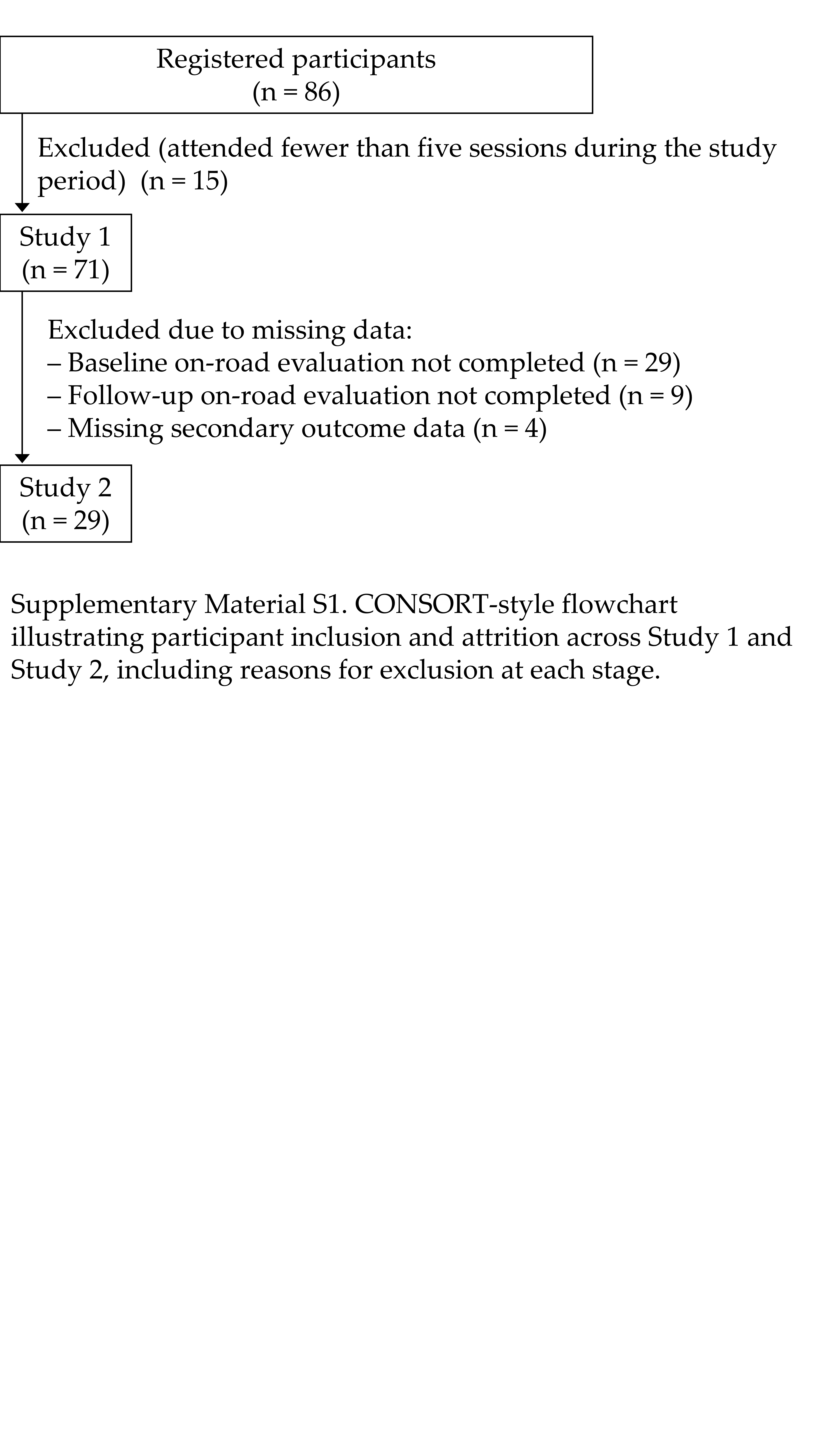

Supplement: Supplementary file 1 [file geriatrics-11-00031-s001.zip › S1&2_revised3/S1_revised3.png]
